# Synthesis of New 1,2,3-Triazole Derivatives of Uracil and Thymine with Potential Inhibitory Activity against Acidic Corrosion of Steels

**DOI:** 10.3390/molecules18044613

**Published:** 2013-04-18

**Authors:** Guillermo E. Negrón-Silva, Rodrigo González-Olvera, Deyanira Angeles-Beltrán, Nidia Maldonado-Carmona, Araceli Espinoza-Vázquez, Manuel E. Palomar-Pardavé, Mario A. Romero-Romo, Rosa Santillan

**Affiliations:** 1 Departamento de Ciencias Básicas, Universidad Autónoma Metropolitana, Av. San Pablo No. 180, México D.F., C.P. 02200, Mexico; E-Mails: rogo@correo.azc.uam.mx (R.G.-O.); dab@correo.azc.uam.mx (D.A.-B.); ponque.chem@gmail.com (N.M.-C.); arasv_21@hotmail.com (A.E.-V.); 2 Departamento de Materiales, Universidad Autónoma Metropolitana, Av. San Pablo No. 180, México D.F., C.P. 02200, Mexico; E-Mails: mepp@correo.azc.uam.mx (M.E.P.-P.); mmrr@correo.azc.uam.mx (M.A.R.-R.); 3 Departamento de Química, Centro de Investigación y de Estudios Avanzados del Instituto Politécnico Nacional, Apartado Postal 14-740, 07000 México D.F., Mexico; E-Mail: rsantill@cinvestav.mx

**Keywords:** synthesis, triazoles, pyrimidines, biological, corrosion

## Abstract

Ten 1,4-disubstituted 1,2,3-triazoles were synthesized from one of 1-(azido-methyl)benzene, 1-(azidomethyl)-4-fluorobenzene, 1-(azidomethyl)-4-chlorobenzene, 1-(azidomethyl)-4-bromobenzene or 1-(azidomethyl)-4-iodobenzene, generated *in situ* from sodium azide and the corresponding benzyl halide, and dipropargyl uracil or dipropargyl thymine. Optimal experimental conditions were established for the conventional click chemistry. The corrosion inhibiting properties of some of these compounds, which were determined by means of an electrochemical technique, are also presented.

## 1. Introduction

Corrosion problems have received considerable attention due to their detrimental effects on materials that contribute to substantial economic losses and environmental pollution. The use of organic inhibitors is one of the most practical and environmentally-friendly methods to protect metals and alloys against corrosion, particularly at acid pHs that certainly damage the steel industrial infrastructure, relevant to overall economic behaviour as one determinant factor of the GNP. A number of heterocyclic organic compounds having either a delocalized set of electrons or just an electron pair on nitrogen, oxygen or sulfur heteroatoms, through which they adsorb on metallic surfaces, can block the active sites to decrease the rate of corrosion of steel, for example. Pyrimidines have various pharmaceutical applications as analgesic, antipyretic, antihypertensive and anti-inflammatory drugs, in pesticides, herbicides, plant growth regulators, and as organic calcium channel modulators [1,2,3,4]. A perusal of the literature revealed that pyrimidine derivatives and the uracil and thymine nitrogen bases [5,6,7,8,9,10,11,12,13,14,15,16] are efficient inhibitors to protect steel in acidic solutions [15,17]. 1,2,3-Triazoles have been the subject of considerable research, mainly due to their usefulness in synthetic organic chemistry and also due to their variety of interesting biological activities, forming part of the scaffolds of antibacterial and antituberculosis agents [18,19], neuraminidase inhibitors [20], anticancer compounds [21,22,23,24], antiviral agents [25,26,27], analgesic compounds [28], fungicidal activity [29,30,31], protein tyrosine phosphatase inhibitors [32,33], and assorted biomolecules (nucleosides and nucleotides) [34,35]. On the other hand, 1,2,3-triazoles have other applications, like in chemosensors [36] and organocatalysts [37]. Just as relevant to applications of 1,2,3-triazoles in the biological and pharmaceutical fields, these molecules belong to the heterocyclic compounds class, constituting an important category of organic inhibitors for corrosion protection of industrial alloys in different acidic solutions. Many substituted triazole compounds continue to be of scientific and engineering interest, and thus they have been studied in considerable detail as effective corrosion inhibitors for steel in acidic media [38,39,40,41,42,43,44,45,46,47,48,49,50,51,52,53,54,55,56,57]. In this context, we decided to explore the synthesis of some new 1,2,3-triazoleuracils and 1,2,3-triazolethymines and their anti-corrosion properties.

## 2. Results and Discussion

### 2.1. Synthesis

The dipropargyl uracil **3** was prepared from uracil (**1**) and propargyl bromide in the presence of DBU by refluxing the mixture in acetonitrile for 18 h. The TLC (CH_2_Cl_2_/MeOH 95:5 *v*/*v*) for the crude reaction mixture showed the formation of **3** (major product) and *N-*1-propargyluracil (minor product). 

**Scheme 1:**
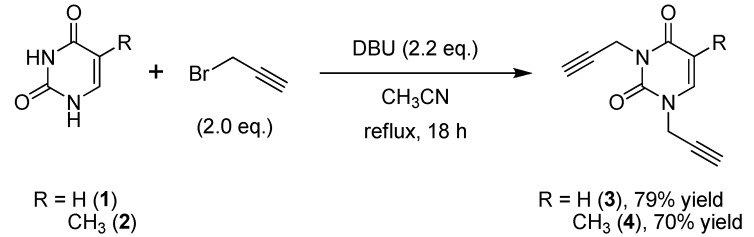
Dialkylation of pyrimidine nucleobases **1**–**2** with propargyl bromide.

The desired product **3** was obtained in 79% yield before purification by column chromatography (Scheme 1). These same conditions were used for the preparation of **4**, which was obtained in 70% yield from thymine (Scheme 1). The second step of the synthesis was the preparation of 1,2,3-triazole nucleobases **5**–**14** through a Huisgen cycloaddition reaction between azides and the terminal alkynes. Based on the Fokin and Van der Eychen methodology [58], it was decided to carry out a multicomponent Cu(I)-catalyzed click reaction for the preparation of the compounds [59,60,61,62,63,64,65,66]. Initially, the reaction was tested by mixing dipropargyl uracil **3**, benzyl chloride and sodium azide in the presence of triethylamine and a catalytic amount of Cu(I) iodide [67,68,69,70,71], and stirring at room temperature for 36 h, whereby compound **5** was obtained in 44% yield (Table 1, entry 1). When the reaction was repeated using Cu(OAc)_2_•H_2_O as precatalyst [72,73,74], sodium ascorbate as reducing agent, and 1,10-phenanthroline•H_2_O as ligand [67,75] in EtOH-H_2_O at room temperature, compound **5** was obtained in 74% yield (Table 1, entry 2). With these reaction conditions established, the remaining compounds **6**–**14** were prepared and obtained in good yields (Table 1, entries 3-11).

**Table 1:** Multicomponent click reaction catalyzed by Cu(OAc)_2_•H_2_O. 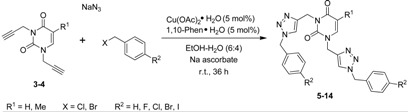

Entry	Compound	R^1^	R^2^	X	Yield ^a^ (%)
1 ^b^	**5**	H	H	Cl	44
2	**5**	H	H	Cl	74
3	**6**	H	F	Cl	78
4	**7**	H	Cl	Cl	71
5	**8**	H	Br	Br	87
6	**9**	H	I	Br	84
7	**10**	CH_3_	H	Cl	64
8	**11**	CH_3_	F	Cl	87
9	**12**	CH_3_	Cl	Cl	74
10	**13**	CH_3_	Br	Br	73
11	**14**	CH_3_	I	Br	86

^a^ Isolated yields after purification; ^b^ The reaction was carried out employing 0.53 mmol of **3**, Cu(I) iodide (5 mol%), and NEt_3_ (3 eq.), stirring at room temperature for 36 h.

The structure of all compounds was confirmed by examination of their ^1^H- and ^13^C-NMR spectra, IR and mass spectra. The presence of the 1,4-disubstituted 1,2,3-triazole nucleobases was unequivocally established by means of the characteristic chemical shift values of the triazolyl hydrogens at 7.49–8.14 ppm and the chemical shift values for the carbon atoms of the triazole ring at 123.4–124.5 ppm for CH and 142.3–143.7 ppm for the quaternary carbon (Table 2). These chemical shift values are completely consistent with those reported by Creary for 1,4-disubstituted 1,2,3-triazoles [76].

**Table 2:** ^1^H- and ^13^C-NMR chemical shifts (ppm) of the triazole ring in compounds **5**–**14**. 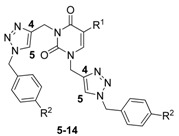

Compound	R^1^	R^2^	H-5	C-5	C-4
**5**	H	H	7.49/7.62	123.4/123.7	142.3/143.4
**6**	H	F	7.96/8.12	124.0/124.3	143.0/143.4
**7**	H	Cl	7.98/8.14	124.2/124.5	143.0/143.4
**8**	H	Br	7.98/8.13	124.2/124.5	143.0/143.4
**9**	H	I	7.95/8.12	124.1/124.5	143.0/143.4
**10**	CH_3_	H	7.49/7.62	123.5/123.7	142.6/143.5
**11**	CH_3_	F	7.96/8.12	124.0/124.3	143.1/143.5
**12**	CH_3_	Cl	7.50/7.64	123.5/123.7	142.8/143.7
**13**	CH_3_	Br	7.50/7.65	123.5/123.7	142.8/143.7
**14**	CH_3_	I	7.95/8.11	124.1/124.4	143.1/143.5

### 2.2. Corrosion Inhibition Efficiencies

**Figure 1:**
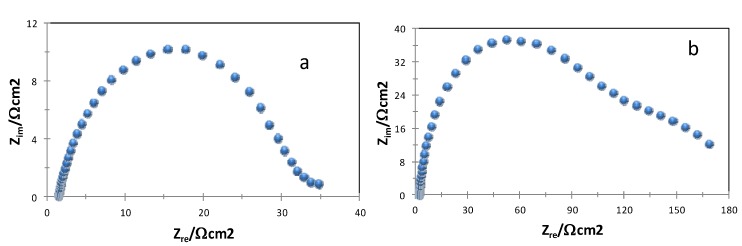
Experimental impedance data, Nyquist plots, recorded in the systems a) API 5L X52/1 M HCl and b) API 5L X52/1 M HCl + 25 ppm of compound **3**.

We evaluated, using electrochemical means, the corrosion inhibition efficiencies of the first four compounds considered in this work. Figure 1 depicts, as an example, the impedance measurements for both bare steel surfaces immersed in 1 M HCl (see Figure 1a), and the same system after adding 25 ppm of compound **3** to the acid media (see Figure 1b). The results of the analysis of the impedance data are shown in Table 3. It is important to remark that all these compounds have corrosion inhibition efficiencies, IE, close to 90% at a rather low concentration value.

**Table 3:** Electrochemical parameters obtained from experimental impedance data, see Figure 1, including the corrosion inhibition efficiencies, IE.

Compound	Rs/Ω cm^2^	Rp/Ω cm^2^	C/µF	IE/%
Blank	2.4	30	310	-
1	2.5	242	94	88
2	7.1	304	97	90
3	2.0	163	167	82
4	1.8	233	100	87

## 3. Experimental

### 3.1. General

Commercially available reagents and solvents were used as received. Flash column chromatography was performed on Kieselgel silica gel 60 (230–400 mesh). Melting points were determined on a Fisher-Johns apparatus and are uncorrected. IR spectra were recorded on a Bruker Alpha FT-IR/ATR spectrometer (Leipzig, Germany). NMR spectra (^1^H at 500 MHz, ^13^C at 125.76 MHz) were obtained with a JEOL ECA-500 (500 MHz) spectrometer (Tokyo, Japan). Chemical shifts (δ) are given in ppm downfield from Me_4_Si used as an internal reference; coupling constants are given in *J* (Hertz). High-resolution mass spectra (HRMS) were recorded on a JEOL JMS-SX 102a and Agilent-MSD-TOF-1069A spectrometers (Tokyo, Japan). The electrochemical impedance study was performed at room temperature using the IM6-ZAHNER electrochemical workstation (ZAHNER-Elektrik GmbH & Co.KG, Kronach, Germany), applying a sinusoidal ± 10 mV perturbation, within the frequency range of 100 KHz to 0.1 Hz to an electrochemical cell with three-electrode setup. A saturated Ag/AgCl mini-electrode was used as reference, with a graphite bar as counter electrode, while the working electrode was a steel sample of API 5L X52 with an exposed area of approximately 1 cm^2^, which was prepared using standard metallographic procedures. The corrosion inhibition efficiency (IE) was evaluated by means of Electrochemical Impedance Spectroscopy (EIS) in the API 5L X52/1 M HCl system containing 0 (blank) or 25 ppm of the inhibitor molecule. Simulation of the impedance data recorded was conducted by means of electrical equivalent circuits [17] and the electrical parameters: Rs, solution resistance, Rtc, charge transfer resistance and C, the capacitance, were obtained in this way.

### 3.2. Product Synthesis and Characterization

*1,3-Di(prop-2-ynyl)pyrimidine-2,4(1H,3H)-dione* (**3**). In a 100 mL round-bottom flask containing a magnetic stirrer and equipped with a reflux condenser, uracil (**1**, 1.12 g, 10 mmol) was suspended in dry acetonitrile (15 mL), 1,8-diazabicyclo[5.4.0]undec-7-ene (DBU, 3.3 mL, 3.35 g, 22 mmol) was added and the mixture stirred for a few minutes until a clear solution was obtained. Subsequently, propargyl bromide (80 wt. % in toluene, 2.22 mL, 2.97 g, 25 mmol) was added and the whole reaction mixture was heated at reflux for 18 h. The acetonitrile was evaporated under vacuum and CH_2_Cl_2_ (20 mL) was added. The organic phase was washed with aqueous NH_4_Cl solution (5%, 20 mL), dried with anhydrous Na_2_SO_4_ and concentrated under vacuum. The residue was purified by column chromatography (CH_2_Cl_2_) and recrystallized from CH_2_Cl_2_/hexane (1:1 *v*/*v*) to obtain 1.48 g (79% yield) of **3** as a white solid, m.p. 102–104 °C [Lit. [77] m.p. 105 °C]. ^1^H-NMR (CDCl_3_): δ = 2.17 (t, *J* = 2.5 Hz, 1H, ≡C-H), 2.50 (t, *J* = 2.6 Hz, 1H, ≡C-H), 4.59 (d, *J* = 2.6 Hz, 2H, CH_2_), 4.68 (d, *J* = 2.5 Hz, 2H, CH_2_), 5.83 (d, *J* = 8.0 Hz, 1H, CH), 7.46 (d, *J* = 8.0 Hz, 1H, NCH). ^13^C-NMR (CDCl_3_): δ = 30.5 (CH_2_), 38.0 (CH_2_), 70.9 (≡C-H), 75.9 (C), 76.0 (≡C-H), 77.9 (C), 102.4 (CH), 140.8 (NCH), 150.5 (N_2_C=O), 161.7 (NC=O). FT-IR/ATR ν_max_ cm^−1^: 3,289, 3,259 (≡C-H), 3,117, 3,090, 3,006, 2,983, 2,949, 2,124 (C≡C), 1,708 (C=C), 1,648 (NC=O, N_2_C=O), 1,540, 1,432. HRMS (ESI-TOF) calculated for C_10_H_8_N_2_O_2_+H^+^: 189.0658; Found: 189.0661.

*5-Methyl-1,3-di(prop-2-ynyl)pyrimidine-2,4-(1H,3H)-dione* (**4**). The procedure described above was followed to obtain compound **4**, employing thymine (**2**, 1.26 g, 10 mmol), DBU (3.3 mL, 22 mmol) and propargyl bromide (80 wt. % in toluene, 2.22 mL, 25 mmol). The crude product was purified by column chromatography (CH_2_Cl_2_) and recrystallized from CH_2_Cl_2_/hexane (1:1 *v*/*v*) to afford 1.41 g (70% yield) of **4** as a white solid, m.p. 96–100 °C [Lit. [77] m.p. 101 °C]. ^1^H-NMR (CDCl_3_): δ = 1.97 (d, *J* = 1.3 Hz, 3H, CH_3_), 2.17 (t, *J* = 2.5 Hz, 1H, ≡C-H), 2.47 (t, *J* = 2.6 Hz, 1H, ≡C-H), 4.57 (d, *J* = 2.6 Hz, 2H, CH_2_), 4.72 (d, *J* = 2.5 Hz, 2H, CH_2_), 7.26 (q, *J* = 1.2 Hz, 1H, NCH). ^13^C-NMR (CDCl_3_): δ = 13.2 (CH_3_), 30.7 (CH_2_), 37.6 (CH_2_), 70.8 (≡C-H), 75.4 (≡C-H), 76.4 (C), 78.1 (C), 110.9 (CCH_3_), 136.8 (NCH), 150.4 (N_2_C=O), 162.6 (NC=O). FT-IR/ATR ν_max_ cm^−1^: 3,274, 3,260 (≡C-H), 3,072, 2,985, 2,931, 2,128 (C≡C), 1,700 (C=C), 1,657 (NC=O), 1,628 (N_2_C=O), 1,463. HRMS (ESI-TOF) calculated for C_11_H_10_N_2_O_2_+H^+^: 203.0815; Found: 203.0817.

*1,3-Bis((1-benzyl-1H-1,2,3-triazol-4-yl)methyl)pyrimidine-2,4-(1H,3H)-dione* (**5**). In a 50 mL round-bottomed flask equipped with a magnetic stirrer, were added Cu(OAc)_2_•H_2_O (5 mg, 0.027 mmol, 5 mol%), 1,10-phenanthroline monohydrate (5 mg, 0.027 mmol, 5 mol%) and sodium L-ascorbate (107 mg, 0.54 mmol) in EtOH-H_2_O (6:4 *v*/v, 5 mL), followed by stirring for five minutes at room temperature. Subsequently, **3** (100 mg, 0.53 mmol), sodium azide (76 mg, 1.17 mmol) and benzyl chloride (0.13 mL, 1.17 mmol) were added to the reaction mixture, which was stirred during 36 h at room temperature (the product starts to precipitate after a few hours). Afterwards, H_2_O (15 mL) was added to the reaction mixture to complete precipitation of the product, which was filtered off, washed with H_2_O, then with hexane and dried under vacuum. The crude product was purified by column chromatography (CH_2_Cl_2_/EtOH 98:2 *v*/*v*) and recrystallized from CH_2_Cl_2_/hexane (1:1 *v*/*v*) to afford 180 mg (74% yield) of **5** as a white solid, m.p. 171–172 °C. ^1^H-NMR (CDCl_3_): δ = 4.93 (s, 2H, CH_2_NC=O), 5.15 (s, 2H, CH_2_NC=O), 5.43 (s, 2H, NCH_2_Ph), 5.47 (s, 2H, NCH_2_Ph), 5.69 (d, *J* = 7.9 Hz, 1H, CH), 7.20–7.28 (m, 4H, ArH), 7.32–7.42 (m, 6H, ArH), 7.44 (d, *J* = 8.0 Hz, 1H, NCH), 7.49 (s, 1H, ArH, triazole), 7.62 (s, 1H, ArH, triazole). ^13^C-NMR (CDCl_3_): δ = 36.2 (CH_2_NC=O), 44.2 (CH_2_NC=O), 54.2 (NCH_2_Ph), 54.4 (NCH_2_Ph), 102.0 (CH), 123.4 (ArCH, triazole), 123.7 (ArCH, triazole), 128.2 (2×ArCH), 128.4 (2×ArCH), 128.8 (ArCH), 129.0 (ArCH), 129.1 (2×ArCH), 129.3 (2×ArCH), 134.2 (C_ipso_), 134.6 (C_ipso_), 142.3 (C_ipso_, triazole), 142.6 (NCH), 143.4 (C_ipso_, triazole), 151.2 (N_2_C=O), 162.6 (NC=O). FT-IR/ATR ν_max_ cm^−^^1^: 3,133, 3,067, 3,012, 2,954, 1,700 (C=C), 1,650 (NC=O, N_2_C=O), 1,555, 1,496, 1,452, 1,434. HRMS (ESI-TOF) calculated for C_24_H_22_N_8_O_2_+H^+^: 455.1938; Found: 455.1939.

*1,3-Bis((1-(4-fluorobenzyl)-1H-1,2,3-triazol-4-yl)methyl)pyrimidine-2,4(1H,3H)-dione* (**6**). The procedure described above (using the same quantities of Cu(OAc)_2_•H_2_O, 1,10-phenanthroline monohydrate, and sodium L-ascorbate) was followed to obtain the compound **6**, employing **3** (100 mg, 0.53 mmol), sodium azide (76 mg, 1.17 mmol) and 4-fluorobenzyl chloride (0.14 mL, 1.17 mmol). The crude product was purified by column chromatography (CH_2_Cl_2_/MeOH 90:10 *v*/*v*) and recrystallized from CH_2_Cl_2_/hexane (1:1 *v*/*v*) to afford 203 mg (78% yield) of **6** as a white solid, m.p. 186–188 °C. ^1^H-NMR (DMSO-*d*_6_): δ = 4.95 (s, 2H, CH_2_NC=O), 4.97 (s, 2H, CH_2_NC=O), 5.48 (s, 2H, NCH_2_Ph), 5.53 (s, 2H, NCH_2_Ph), 5.73 (d, *J* = 7.9 Hz, 1H, CH), 7.13–7.19 (m, 4H, ArH), 7.31–7.37 (m, 4H, ArH), 7.80 (d, *J* = 7.9 Hz, 1H, NCH), 7.96 (s, 1H, ArH, triazole), 8.12 (s, 1H, ArH, triazole). ^13^C-NMR (DMSO-*d_6_*): δ = 36.3 (CH_2_NC=O), 44.1 (CH_2_NC=O), 52.4 (NCH_2_Ph), 52.5 (NCH_2_Ph), 101.0 (CH), 116.0 (d, *J*^2^_CF_ = 21.4 Hz, 2×ArCH), 116.2 (d, *J*^2^_CF_ = 21.4 Hz, 2×ArCH), 124.0 (ArCH, triazole), 124.3 (ArCH, triazole), 130.8 (d, *J*^3^_CF_ = 8.8 Hz, 2×ArCH), 130.9 (d, *J*^3^_CF_ = 8.8 Hz, 2×ArCH), 132.6 (d, *J*^4^_CF_ = 2.5 Hz, C_ipso_), 132.8 (d, *J*^4^_CF_ = 2.5 Hz, C_ipso_), 143.0 (C_ipso_, triazole), 143.4 (C_ipso_, triazole), 144.9 (NCH), 151.3 (N_2_C=O), 161.4 (d, *J*_CF_ = 243.8 Hz, C_ipso_), 162.5 (NC=O), 163.4 (d, *J*_CF_ = 243.8 Hz, C_ipso_). FT-IR/ATR ν_max_ cm^−^^1^: 3,126, 3,066, 3,012, 2,969, 1,705 (C=C), 1,657 (NC=O, N_2_C=O), 1,604, 1,544, 1,508, 1,453. HRMS (ESI-TOF) calculated for C_24_H_20_F_2_N_8_O_2_+H^+^: 491.1750; Found: 491.1751.

*1,3-Bis((1-(4-chlorobenzyl)-1H-1,2,3-triazol-4-yl)methyl)pyrimidine-2,4(1H,3H)-dione* (**7**). The procedure described above (using the same quantities of Cu(OAc)_2_•H_2_O, 1,10-phenanthroline monohydrate, and sodium L-ascorbate) was followed to obtain compound **7**, employing **3** (100 mg, 0.53 mmol), NaN_3_ (76 mg, 1.17 mmol) and 4-chlorobenzyl chloride (198 mg, 1.23 mmol). The crude product was purified by column chromatography (CH_2_Cl_2_/EtOH 97:3 *v*/*v*) and recrystallized from CH_2_Cl_2_/hexane (1:1 *v*/*v*) to afford 197 mg (71% yield) of the desired product **7** as a white solid, m.p. 185–187 °C. ^1^H-NMR (DMSO-*d_6_*): δ = 4.96 (s, 2H, CH_2_NC=O), 4.98 (s, 2H, CH_2_NC=O), 5.51 (s, 2H, NCH_2_Ph), 5.55 (s, 2H, NCH_2_Ph), 5.73 (d, *J* = 7.9 Hz, 1H, CH), 7.28 (d, *J* = 8.4 Hz, 2H, ArH), 7.30 (d, *J* = 8.3 Hz, 2H, ArH), 7.39 (d, *J* = 8.4 Hz, 2H, ArH), 7.40 (d, *J* = 8.4 Hz, 2H, ArH), 7.80 (d, *J* = 7.9 Hz, 1H, NCH), 7.98 (s, 1H, ArH, triazole), 8.14 (s, 1H, ArH, triazole). ^13^C-NMR (DMSO-*d_6_*): δ = 36.3 (CH_2_NC=O), 44.1 (CH_2_NC=O), 52.4 (NCH_2_Ph), 52.6 (NCH_2_Ph), 101.0 (CH), 124.2 (ArCH, triazole), 124.5 (ArCH, triazole), 129.27 (2×ArCH), 129.30 (2×ArCH), 130.46 (2×ArCH), 130.49 (2×ArCH), 133.38 (C_ipso_), 133.44 (C_ipso_), 135.4 (C_ipso_), 135.5 (C_ipso_), 143.0 (C_ipso_, triazole), 143.4 (C_ipso_, triazole), 144.9 (NCH), 151.3 (N_2_C=O), 162.6 (NC=O). FT-IR/ATR ν_max_ cm^−^^1^: 3,128, 3,064, 3,013, 2,970, 2,951, 1,702 (C=C), 1,655 (NC=O, N_2_C=O), 1,542, 1,491, 1,453. HRMS (ESI-TOF) calculated for C_24_H_20_Cl_2_N_8_O_2_+H^+^: 523.1159; Found: 523.1158.

*1,3-Bis((1-(4-bromobenzyl)-1H-1,2,3-triazol-4-yl)methyl)pyrimidine-2,4(1H,3H)-dione* (**8**). The procedure described above (using the same quantities of Cu(OAc)_2_•H_2_O, 1,10-phenanthroline monohydrate, sodium L-ascorbate) was followed to obtain compound **8**, employing **3** (100 mg, 0.53 mmol), NaN_3_ (76 mg, 1.17 mmol) and 4-bromobenzyl bromide (292 mg, 1.17 mmol). The crude product was purified by column chromatography (CH_2_Cl_2_/EtOH 97:3 *v*/*v*) and recrystallized from CH_2_Cl_2_/hexane (1:1 *v*/*v*) to afford 288 mg (87% yield) of the desired product **8** as a white solid, m.p. 196–198 °C. ^1^H-NMR (DMSO-*d_6_*): δ = 4.96 (s, 2H, CH_2_NC=O), 4.98 (s, 2H, CH_2_NC=O), 5.49 (s, 2H, NCH_2_Ph), 5.53 (s, 2H, NCH_2_Ph), 5.73 (d, *J* = 7.9 Hz, 1H, CH), 7.21 (d, *J* = 8.4 Hz, 2H, ArH), 7.23 (d, *J* = 8.3 Hz, 2H, ArH), 7.53 (d, *J* = 8.3 Hz, 2H, ArH), 7.54 (d, *J* = 8.4 Hz, 2H, ArH), 7.80 (d, *J* = 7.9 Hz, 1H, NCH), 7.98 (s, 1H, ArH, triazole), 8.13 (s, 1H, ArH, triazole). ^13^C-NMR (DMSO-*d_6_*): δ = 36.3 (CH_2_NC=O), 44.1 (CH_2_NC=O), 52.5 (NCH_2_Ph), 52.6 (NCH_2_Ph), 101.0 (CH), 121.9 (C_ipso_), 122.0 (C_ipso_), 124.2 (ArCH, triazole), 124.5 (ArCH, triazole), 130.76 (2×ArCH), 130.79 (2×ArCH), 132.21 (2×ArCH), 132.23 (2×ArCH), 135.8 (C_ipso_), 135.9 (C_ipso_), 143.0 (C_ipso_, triazole), 143.4 (C_ipso_, triazole), 144.9 (NCH), 151.2 (N_2_C=O), 162.5 (NC=O). FT-IR/ATR ν_max_ cm^−^^1^: 3,141, 3,128, 3,073, 1,703 (C=C), 1,656 (NC=O, N_2_C=O), 1,593, 1,543, 1,488, 1,455. HRMS (ESI-TOF) calculated for C_24_H_20_Br_2_N_8_O_2_+H^+^: 611.0148; Found: 611.0150.

*1,3-Bis((1-(4-iodobenzyl)-1H-1,2,3-triazol-4-yl)methyl)pyrimidine-2,4(1H,3H)-dione* (**9**). The procedure described above (using the same quantities of Cu(OAc)_2_•H_2_O, 1,10-phenanthroline mono-hydrate, sodium L-ascorbate) was followed to obtain compound **9**, employing **3** (100 mg, 0.53 mmol), NaN_3_ (76 mg, 1.17 mmol) and 4-iodobenzyl bromide (365 mg, 1.23 mmol). The crude product was purified by column chromatography (CH_2_Cl_2_/MeOH 90:10 *v*/*v*) and recrystallized from CH_2_Cl_2_/hexane (1:1 *v*/*v*) to afford 315 mg (84% yield) of the desired product **9** as a white solid, m.p. 202–204 °C. ^1^H-NMR (DMSO-*d*_6_): δ = 4.95 (s, 2H, CH_2_NC=O), 4.97 (s, 2H, CH_2_NC=O), 5.46 (s, 2H, NCH_2_Ph), 5.50 (s, 2H, NCH_2_Ph), 5.73 (d, *J* = 7.9 Hz, 1H, CH), 7.06 (d, *J* = 8.2 Hz, 2H, ArH), 7.07 (d, *J* = 8.2 Hz, 2H, ArH), 7.69 (d, *J* = 8.2 Hz, 2H, ArH), 7.70 (d, *J* = 8.3 Hz, 2H, ArH), 7.79 (d, *J* = 7.9 Hz, 1H, NCH), 7.95 (s, 1H, ArH, triazole), 8.12 (s, 1H, ArH, triazole). ^13^C-NMR (DMSO-*d_6_*): δ = 36.3 (CH_2_NC=O), 44.1 (CH_2_NC=O), 52.6 (NCH_2_Ph), 52.8 (NCH_2_Ph), 95.02 (C_ipso_), 95.08 (C_ipso_), 101.0 (CH), 124.1 (ArCH, triazole), 124.5 (ArCH, triazole), 130.81 (2×ArCH), 130.83 (2×ArCH), 136.2 (C_ipso_), 136.3 (C_ipso_), 138.06 (2×ArCH), 138.08 (2×ArCH), 143.0 (C_ipso_, triazole), 143.4 (C_ipso_, triazole), 144.9 (NCH), 151.2 (N_2_C=O), 162.5 (NC=O). FT-IR/ATR ν_max_ cm^−^^1^: 3,127, 3,074, 3,011, 2,944, 1,703 (C=C), 1,654 (NC=O, N_2_C=O), 1,588, 1,544, 1,484, 1,453. HRMS (ESI-TOF) calculated for C_24_H_20_I_2_N_8_O_2_+H^+^: 706.9871; Found: 706.9851. 

*1,3-Bis((1-benzyl-1H-1,2,3-triazol-4-yl)methyl)-5-methylpyrimidine-2,4(1H,3H)-dione* (**10**). The procedure described above was followed to obtain compound **10**, employing Cu(OAc)_2_•H_2_O (4.5 mg, 0.025 mmol), 1,10-phenanthroline monohydrate (5 mg, 0.025 mmol), sodium L-ascorbate (99 mg, 0.5 mmol), **4** (100 mg, 0.49 mmol), NaN_3_ (70 mg, 1.08 mmol) and benzyl chloride (0.12 mL, 1.08 mmol). The crude product was purified by column chromatography (CH_2_Cl_2_/EtOH 98:2 *v*/*v*) and recrystallized from CH_2_Cl_2_/hexane (1:1 *v*/*v*) to afford 148 mg (64% yield) of the desired product **10** as a white solid, m.p. 187–189 °C. ^1^H-NMR (CDCl_3_): δ = 1.86 (d, *J* = 1.0 Hz, 3H, CH_3_), 4.91 (s, 2H, CH_2_NC=O), 5.16 (s, 2H, CH_2_NC=O), 5.43 (s, 2H, NCH_2_Ph), 5.46 (s, 2H, NCH_2_Ph), 7.21–7.28 (m, 4H, ArH), 7.29 (d, *J* = 1.1 Hz, 1H, NCH), 7.30–7.37 (m, 6H, ArH), 7.49 (s, 1H, ArH, triazole), 7.62 (s, 1H, ArH, triazole). ^13^C-NMR (CDCl_3_): δ = 13.0 (CH_3_), 36.4 (CH_2_NC=O), 43.9 (CH_2_NC=O), 54.2 (NCH_2_Ph), 54.4 (NCH_2_Ph), 110.3 (CCH_3_), 123.5 (ArCH, triazole), 123.7 (ArCH, triazole), 128.2 (2×ArCH), 128.4 (2×ArCH), 128.8 (ArCH), 129.0 (ArCH), 129.1 (2×ArCH), 129.3 (2×ArCH), 134.3 (C_ipso_), 134.7 (C_ipso_), 138.7 (NCH), 142.6 (C_ipso_, triazole), 143.5 (C_ipso_, triazole), 151.2 (N_2_C=O), 163.5 (NC=O). FT-IR/ATR ν_max_ cm^−^^1^: 3,133, 3,115, 3,068, 2,956, 1,697 (C=C), 1,672 (NC=O), 1,646 (N_2_C=O), 1,550, 1,496, 1,453, 1,432. HRMS (ESI-TOF) calculated for C_25_H_24_N_8_O_2_+H^+^: 469.2094; Found: 469.2096.

*1,3-Bis((1-(4-fluorobenzyl)-1H-1,2,3-triazol-4-yl)methyl)-5-methylpyrimidine-2,4(1H,3H)-dione* (**11**). The procedure described above was followed to obtain compound **11**, employing Cu(OAc)_2_•H_2_O (4.5 mg, 0.025 mmol), 1,10-phenanthroline monohydrate (5 mg, 0.025 mmol), sodium L-ascorbate (99 mg, 0.5 mmol), **4** (100 mg, 0.49 mmol), NaN_3_ (70 mg, 1.08 mmol) and 4-fluorobenzyl chloride (0.13 mL, 1.08 mmol). The crude product was purified by column chromatography (CH_2_Cl_2_/MeOH 90:10 *v*/*v*) and recrystallized from CH_2_Cl_2_/hexane (1:1 *v*/*v*) to afford 216 mg (87% yield) of the desired product **11** as a white solid, m.p. 170–172 °C. ^1^H-NMR (DMSO-*d*_6_): δ = 1.77 (d, *J* = 1.0 Hz, 3H, CH_3_), 4.92 (s, 2H, CH_2_NC=O), 4.99 (s, 2H, CH_2_NC=O), 5.49 (s, 2H, NCH_2_Ph), 5.53 (s, 2H, NCH_2_Ph), 7.13–7.19 (m, 4H, ArH), 7.31–7.37 (m, 4H, ArH), 7.69 (d, *J* = 1.1 Hz, 1H, NCH), 7.96 (s, 1H, ArH, triazole), 8.12 (s, 1H, ArH, triazole). ^13^C-NMR (DMSO-*d_6_*): δ = 13.1 (CH_3_), 36.6 (CH_2_NC=O), 44.0 (CH_2_NC=O), 52.4 (NCH_2_Ph), 52.5 (NCH_2_Ph), 108.6 (CCH_3_), 116.0 (d, *J*^2^_CF_ = 21.4 Hz, 2×ArCH), 116.2 (d, *J*^2^_CF_ = 21.4 Hz, 2×ArCH), 124.0 (ArCH, triazole), 124.3 (ArCH, triazole), 130.8 (d, *J*^3^_CF_ = 8.8 Hz, 2×ArCH), 130.9 (d, *J*^3^_CF_ = 8.8 Hz, 2×ArCH), 132.7 (d, *J*^4^_CF_ = 2.5 Hz, C_ipso_), 132.8 (d, *J*^4^_CF_ = 2.5 Hz, C_ipso_), 140.7 (NCH), 143.1 (C_ipso_, triazole), 143.5 (C_ipso_, triazole), 151.1 (N_2_C=O), 161.4 (d, *J*_CF_ = 245.1 Hz, C_ipso_), 163.3 (NC=O), 163.4 (d, *J*_CF_ = 245.1 Hz, C_ipso_). FT-IR/ATR ν_max_ cm^−^^1^: 3,133, 3,073, 3,012, 2,954, 1,694, 1,664, 1,641, 1,605, 1,510, 1,463, 1,435. HRMS (ESI-TOF) calculated for C_25_H_22_F_2_N_8_O_2_+H^+^: 505.1906; Found: 505.1916.

*1,3-Bis((1-(4-chlorobenzyl)-1H-1,2,3-triazol-4-yl)methyl)-5-methylpyrimidine-2,4-(1H,3H)-dione* (**12**). The procedure described above was followed to obtain compound **12**, employing 4.5 mg (0.025 mmol) of Cu(OAc)_2_•H_2_O, 1,10-phenanthroline monohydrate (5 mg, 0.025 mmol), sodium L-ascorbate (99 mg, 0.5 mmol), **4** (100 mg, 0.49 mmol), NaN_3_, (70 mg, 1.08 mmol) and 4-chlorobenzyl chloride (184 mg, 1.14 mmol). The crude product was purified by column chromatography (CH_2_Cl_2_/EtOH 97:3 *v*/*v*) and recrystallized from CH_2_Cl_2_/hexane (1:1 *v*/*v*) to afford 198 mg (74% yield) of the desired product **12** as a white solid, m.p. 154–156 °C. ^1^H-NMR (CDCl_3_): δ = 1.87 (d, *J* = 1.1 Hz, 3H, CH_3_), 4.91 (s, 2H, CH_2_NC=O), 5.17 (s, 2H, CH_2_NC=O), 5.41 (s, 2H, NCH_2_Ph), 5.44 (s, 2H, NCH_2_Ph), 7.17 (d, *J* = 8.5 Hz, 2H, ArH), 7.21 (d, *J* = 8.5 Hz, 2H, ArH), 7.29 (d, *J* = 1.1 Hz, 1H, NCH), 7.30 (d, *J* = 8.5 Hz, 2H, ArH), 7.33 (d, *J* = 8.5 Hz, 2H, ArH), 7.50 (s, 1H, ArH, triazole), 7.64 (s, 1H, ArH, triazole). ^13^C-NMR (CDCl_3_): δ = 13.0 (CH_3_), 36.3 (CH_2_NC=O), 44.1 (CH_2_NC=O), 53.4 (NCH_2_Ph), 53.6 (NCH_2_Ph), 110.3 (CCH_3_), 123.5 (ArCH, triazole), 123.7 (ArCH, triazole), 129.3 (2×ArCH), 129.5 (2×ArCH), 129.6 (2×ArCH), 129.7 (2×ArCH), 132.8 (C_ipso_), 133.1 (C_ipso_), 134.8 (C_ipso_), 135.1 (C_ipso_), 138.7 (NCH), 142.8 (C_ipso_, triazole), 143.7 (C_ipso_, triazole), 151.2 (N_2_C=O), 163.5 (NC=O). FT-IR/ATR ν_max_ cm^−^^1^: 3,142, 3,121, 3,068, 3,012, 2,955, 2,925, 1,693 (C=C), 1,662 (NC=O), 1,637 (N_2_C=O), 1,491, 1,462, 1,433. HRMS (ESI-TOF) calculated for C_25_H_22_Cl_2_N_8_O_2_+H^+^: 537.1315; Found: 537.1323.

*1,3-Bis((1-(4-bromobenzyl)-1H-1,2,3-triazol-4-yl)methyl)-5-methylpyrimidine-2,4-(1H,3H)-dione* (**13**). The procedure described above was followed to obtain compound **13**, employing Cu(OAc)_2_•H_2_O (4.5 mg, 0.025 mmol), 1,10-phenanthroline monohydrate (5 mg, 0.025 mmol), sodium L-ascorbate (99 mg, 0.5 mmol), **4** (100 mg, 0.49 mmol), NaN_3_ (70 mg, 1.08 mmol) and 4-bromobenzyl bromide (270 mg, 1.08 mmol). The crude product was purified by column chromatography (CH_2_Cl_2_/EtOH 97:3 *v*/*v*) and recrystallized from CH_2_Cl_2_/hexane (1:1 *v*/*v*) to afford 225 mg (73% yield) of the desired product **13** as a white solid, m.p. 148–150 °C. ^1^H-NMR (CDCl_3_): δ = 1.87 (d, *J* = 1.2 Hz, 3H, CH_3_), 4.91 (s, 2H, CH_2_NC=O), 5.16 (s, 2H, CH_2_NC=O), 5.39 (s, 2H, NCH_2_Ph), 5.42 (s, 2H, NCH_2_Ph), 7.10 (d, *J* = 8.6 Hz, 2H, ArH), 7.15 (d, *J* = 8.6 Hz, 2H, ArH), 7.29 (d, *J* = 1.2 Hz, 1H, NCH), 7.46 (d, *J* = 8.5 Hz, 2H, ArH), 7.49 (d, *J* = 8.5 Hz, 2H, ArH), 7.50 (s, 1H, ArH, triazole), 7.65 (s, 1H, ArH, triazole). ^13^C-NMR (CDCl_3_): δ = 13.0 (CH_3_), 36.3 (CH_2_NC=O), 44.1 (CH_2_NC=O), 53.5 (NCH_2_Ph), 53.7 (NCH_2_Ph), 110.3 (CCH_3_), 122.9 (C_ipso_), 123.2 (C_ipso_), 123.5 (ArCH, triazole), 123.7 (ArCH, triazole), 129.9 (2×ArCH), 130.0 (2×ArCH), 132.3 (2×ArCH), 132.4 (2×ArCH), 133.3 (C_ipso_), 133.7 (C_ipso_), 138.7 (NCH), 142.8 (C_ipso_, triazole), 143.7 (C_ipso_, triazole), 151.2 (N_2_C=O), 163.4 (NC=O). FT-IR/ATR ν_max_ cm^−^^1^: 3,139, 3,101, 3,069, 2,998, 2,953, 2,922, 1,693 (C=C), 1,661 (NC=O), 1,637 (N_2_C=O), 1,488, 1,462, 1,432. HRMS (ESI-TOF) calculated for C_25_H_22_Br_2_N_8_O_2_+H^+^: 625.0305; Found: 625.0311.

*1,3-Bis((1-(4-iodobenzyl)-1H-1,2,3-triazol-4-yl)methyl)-5-methylpyrimidine-2,4-(1H,3H)-dione* (**14**). The procedure described above was followed to obtain compound **14**, employing Cu(OAc)_2_•H_2_O (4.5 mg, 0.025 mmol), 1,10-phenanthroline monohydrate (5 mg (0.025 mmol), sodium L-ascorbate (99 mg, 0.5 mmol), **4** (100 mg, 0.49 mmol), NaN_3_ (70 mg, 1.08 mmol) and 4-iodobenzyl bromide (339 mg, 1.14 mmol). The crude product was purified by column chromatography (CH_2_Cl_2_/MeOH 90:10 *v*/*v*) and recrystallized from CH_2_Cl_2_/hexane (1:1 *v*/*v*) to afford 305 mg (86% yield) of the desired product **14** as a white solid, m.p. 229–231 °C. ^1^H-NMR (DMSO-*d*_6_): δ = 1.77 (s, 3H, CH_3_), 4.92 (s, 2H, CH_2_NC=O), 4.99 (s, 2H, CH_2_NC=O), 5.46 (s, 2H, NCH_2_Ph), 5.50 (s, 2H, NCH_2_Ph), 7.06 (d, *J* = 7.7 Hz, 2H, ArH), 7.07 (d, *J* = 7.7 Hz, 2H, ArH), 7.68–7.71 (m, 5H, NCH, ArH), 7.95 (s, 1H, ArH, triazole), 8.11 (s, 1H, ArH, triazole). ^13^C-NMR (DMSO-*d_6_*): δ = 13.1 (CH_3_), 36.6 (CH_2_NC=O), 44.0 (CH_2_NC=O), 52.6 (NCH_2_Ph), 52.8 (NCH_2_Ph), 95.01 (C_ipso_), 95.07 (C_ipso_), 108.6 (CCH_3_), 124.1(ArCH, triazole), 124.4 (ArCH, triazole), 130.81 (2×ArCH), 130.82 (2×ArCH), 136.2 (C_ipso_), 136.3 (C_ipso_), 138.05 (2×ArCH), 138.08 (2×ArCH), 140.7 (NCH), 143.1 (C_ipso_, triazole), 143.5 (C_ipso_, triazole), 151.1 (N_2_C=O), 163.3 (NC=O). FT-IR/ATR ν_max_ cm^−^^1^: 3,138, 3,119, 3,072, 2,957, 1,696, 1,640, 1,590, 1,555, 1,464. HRMS (ESI-TOF) calculated for C_25_H_22_I_2_N_8_O_2_+H^+^: 721.0028; Found: 721.0019.

## 4. Conclusions

Ten new triazole derivatives of the pyrimidine bases uracil and thymine which are potential corrosion inhibitors of steel in acidic media were synthesized in good yields, employing a “click” multicomponent reaction. 
